# Effect of pharmacological treatment on outcomes of heart failure with preserved ejection fraction: an updated systematic review and network meta-analysis of randomized controlled trials

**DOI:** 10.1186/s12933-022-01679-2

**Published:** 2022-11-08

**Authors:** Yaowang Lin, Zhigang Cai, Jie Yuan, Huadong Liu, Xinli Pang, Qiuling Chen, Xinzheng Tang, Qingshan Geng, Shaohong Dong

**Affiliations:** 1 Department of Cardiology, Shenzhen People’s Hospital (The Second Clinical Medical College, Jinan University; the First Affiliated Hospital, Southern University of Science and Technology), Cardiovascular Minimally Invasive Medical Engineering Technology Research and Development Center, Shenzhen Key Medical Discipline (SZXK003), Shenzhen, 518020 People’s Republic of China; 2 Institution of Shenzhen Hospital, Guangzhou University of Chinese Medicine (Futian), Shenzhen, 518000 People’s Republic of China; 3 Department of Pharmacy, Shenzhen People’s Hospital, Shenzhen, People’s Republic of China; 4 Department of Geriatrics, Shenzhen People’s Hospital, Shenzhen, People’s Republic of China

**Keywords:** Heart failure with preserved ejection fraction, All-cause death, Cardiac death, HF hospitalization, Worsening HF events, Randomized control trials, Registered in PROSPERO (CRD42021247034)

## Abstract

**Background:**

Optimal treatment strategies for patients with heart failure with preserved ejection fraction (HFpEF) remain uncertain. The goal of this study was to compare the treatment effects of different therapeutic agents for patients with HFpEF.

**Methods:**

Randomized controlled trials (RCTs) published before June 2022 were searched from PubMed, Clinical Trials gov, and the Cochrane Central Register databases. Combined odds ratios (ORs) with 95% confidence intervals (CI) were calculated for the primary and secondary outcomes. All-cause death was the primary endpoint and cardiac death, hospitalization for HF, and worsening HF (WHF) events were secondary endpoints in this meta-analysis.

**Results:**

Fifteen RCTs including 31,608 patients were included in this meta-analysis. All-cause and cardiac death were not significantly correlated between drug treatments and placebo. Compared with placebo, angiotensin-converting enzyme inhibitors (ACEIs), angiotensin receptor neprilysin inhibitors (ARNIs), and sodium-glucose cotransporter-2 (SGLT2) inhibitors significantly reduced HF hospitalizations [odds ratio (OR) = 0.64, (95% confidence interval (95%CI 0.43 − 0.96), OR = 0.73, (95%CI 0.61 − 0.86), and OR = 0.74, (95%CI 0.66 − 0.83), respectively] without heterogeneity among studies. Only SGLT2 inhibitors significantly reduced WHF events [OR = 0.75, (95%CI 0.67 − 0.83)].

**Conclusions:**

No treatments were effective in reducing mortality, but ARNIs, ACEIs or SGLT2 inhibitors reduced HF hospitalizations and only SGLT2 inhibitors reduced WHF events for patients with HFpEF.

**Supplementary Information:**

The online version contains supplementary material available at 10.1186/s12933-022-01679-2.

## Background

Heart failure (HF) is associated with substantially high morbidity and mortality as well as high rates of rehospitalization [1, 2]. HF with preserved ejection (HFpEF) has evolved into a major type of HF [3, 4]. HFpEF was previously defined as HF accompanied by left ventricular ejection fraction (LVEF) > 40%, but is currently defined as LVEF > 50% with no history of improved LVEF from < 40% [5]. Mortality (defined as 1-year mortality of approximately 10 − 30% and 5-year mortality of > 50%) and readmission rates of patients with HFpEF are higher compared to those patients with HF with reduced ejection fraction (HFrEF) [6]. Due to the pathophysiological heterogeneity of HFpEF, there is a lack of therapeutic agents that effectively treat these outcomes, which presents a major clinical challenge for patients with HFpEF.

The 2021 European Society of Cardiology guidelines for the diagnosis and treatment of acute and chronic HF recommend the use of diuretics, angiotensin-converting enzyme inhibitors (ACEI), angiotensin receptor neprilysin inhibitors (ARNI), and mineralocorticoid receptor antagonists (MRA) for HFpEF. However, there is a lack of convincing evidence for the ability of these treatments to reduce mortality7. Recently, the EMPEROR-Preserved and the DELIVER study showed that sodium-glucose cotransporter-2 (SGLT2) inhibitors (empagliflozin and dapagliflozin) have a positive effect on composite outcomes in HFpEF [8, 9]. Accordingly, we conducted a network meta-analysis to compare the effects of these drugs with placebo for the treatment of HFpEF.

## Materials

### Search strategy

PubMed, Clinical Trial gov, and the Cochrane Central Register were searched to identify articles published before May 30, 2022 (Additional file 1: Tables S1, S2, S3). The MeSH terms included “heart failure with preserved ejection fraction”, “diastolic heart failure”, “angiotensin receptor neprilysin inhibitor or sacubitril-valsartan”, “angiotensin converting enzyme inhibitors”, “angiotensin receptor blockers”, “beta blockers”, “mineralocorticoid receptor antagonists”, “digoxin”, “phosphodiesterase-5 inhibition or sidenafi”, “vericiguat”, “sodium-glucose cotransporter-2”, and “diuretic”.

### Eligibility criteria

The inclusion criteria were as follows: (i) types of studies: randomized controlled trials (RCTs); (ii) types of participants: HF with LVEF ≥ 40%; (iii) types of interventions: treatment groups received oral drugs; (iv) types of comparators: placebo or no drugs; (v) types of outcome measures: data on all-cause and cardiac mortality, HF hospitalizations, or worsening HF (WHF) events; and (vi) articles published in English. Exclusion criteria were as follows: (i) duplicate publications; (ii) subgroup studies; and (iii) lack of data on endpoint.

### Study outcomes

All-cause death was the primary endpoint, and cardiac death, HF hospitalization, and WHF events (defined as deterioration in heart failure symptoms and signs requiring an intensification of therapy) were the secondary endpoints.

### Data extraction and quality assessment

Data were extracted independently by two authors (Y.W.L. and Z.G.C.) through a detailed review of the full text. Any disagreement between the two researchers was discussed and decided by a third author (S.H.D.). We assessed the risk of bias, publication bias, and overall quality of the literature using the Cochrane Collaboration tool, funnel plots, and confidence in network meta-analysis (CINeMA), respectively.

### Statistical analysis

We used STATA software (version 16.0) for statistical analysis. Pooled odds ratios (OR) and their 95% confidence intervals (CI) were adopted to assess endpoint events. The surface under the cumulative ranking (SUCRA) curve was used to assess the best treatment strategy. Heterogeneity between studies was analyzed using the *I*^*2*^ statistic.

## Results

### Characteristics of included studies

**Fig. 1:**
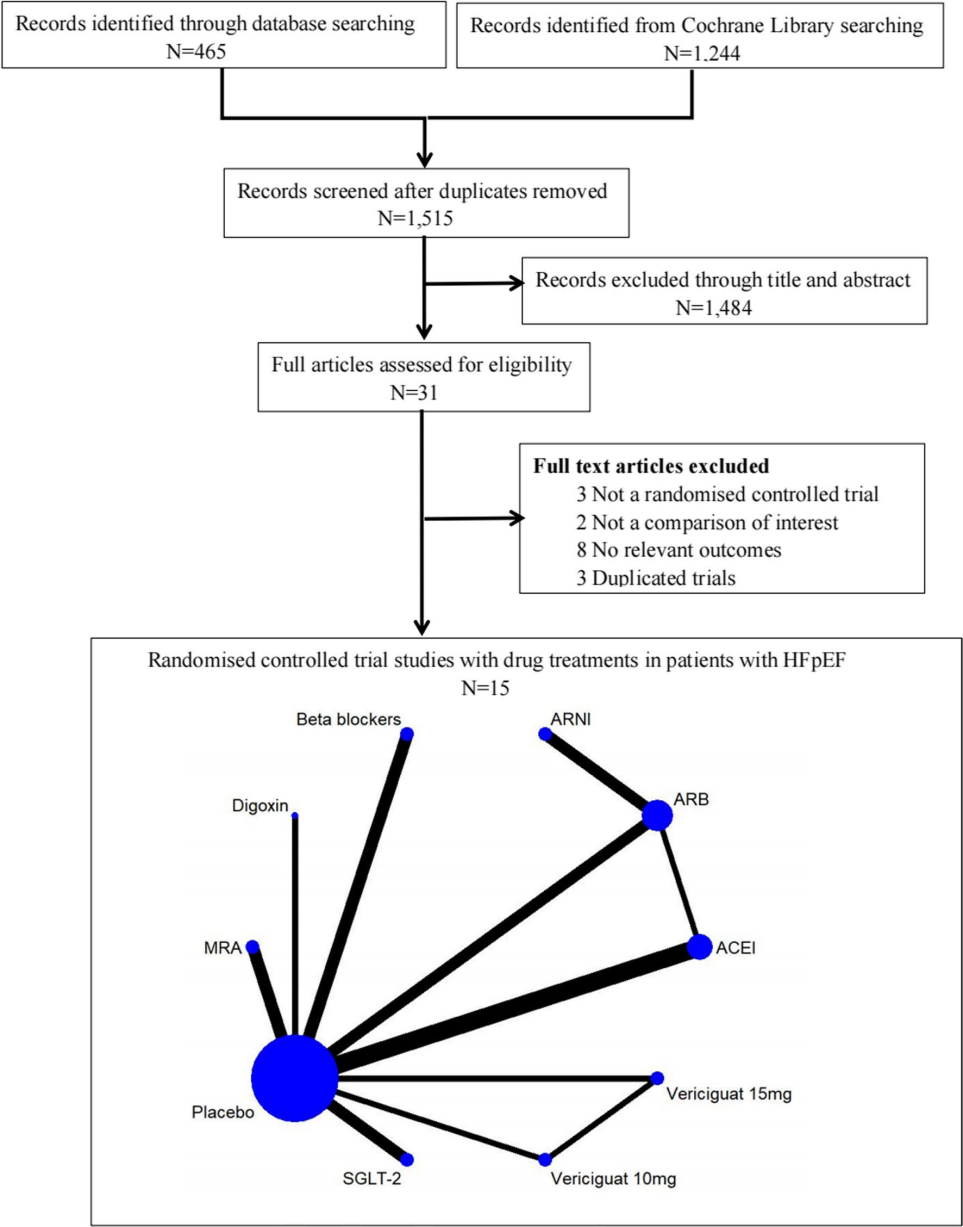
Flow diagram of the study selection process *HF* heart failure, *MRA* mineralocorticoid receptor antagonist, *SLGT2* sodium-glucose cotransporter-2, *ACEI* angiotensin-converting enzyme inhibitor, *ARB* angiotensin receptor blocker, ARNI angiotensin receptor neprilysin inhibitor, *MRA* mineralocorticoid receptor antagonist

A total of 1709 related articles were identified through the database search, of which 168 articles were duplicates. After reading the titles and abstracts, 1214 articles were removed because they did not meet the inclusion criteria. In addition, 16 articles were removed due to lack of endpoint data. Ultimately, 15 RCTs [9–23] (Fig. 1) were included in this meta-analysis. Eight were double-blind, two were single-blind, and five were open-label studies. Finally, 31,608 patients were included (15,969 in the intervention group; 15,639 in the control group) with a follow-up period of 0.5 to 4 years. All patients enrolled were elderly (mean age 66.7 to 89 years) without differences in baseline profiles (Table 1).

**Table 1:** Baseline characteristics of included RCTs

Study	Year/country	Study	Intervention group	Control group	Design	Age, y	NYHA III-IV, %	All-cause mortality	Cardiovascular mortality	Heart failure hospitalization	Worsening heart failure events	Follow-up
Aronow WS	1997 USA	Open-label	Propranolol (n = 79)	Placebo (n = 79)	LVEF ≥ 40%, > 62 years age	81 ± 8 vs. 81 ± 7	47 vs. 49	44/79 vs. 60/797	/	/	/	35 months
Yusuf, S	2003 Canada	Double-blind	Candesartan (n = 1514)	Placebo (n = 1509)	CHARM preserved trial, LVEF ≥ 40%	67.2 ± 11.1 vs 67.1 ± 11.1	38.5 vs. 40	/	170/1514 vs. 170/1509	241/1514vs.276/1509	/	36.6 months
Zi M	2003 UK	Double-blind	Quinapril (n = 36)	Placebo (n = 38)	LVEF ≥ 40%, > 62 years age	77 ± 7 vs. 78 ± 7	16.7 vs. 26.3	1/36 vs. 1/38	1/36 vs. 1/38	2/36 vs. 5/38	0/36 vs. 4/38	6 months
Cleland, JG	2006 UK	Double-blind	perindopril (n = 424)	Placebo (n = 426)	PEP-CHF, LVEF ≥ 40%, > 70 years age	75 (72,79) vs. 75 (72,79)	23 vs. 26	17/424 vs. 19 /4267	10/424 vs. 17/426	34/424 vs. 53 /426	59/424 vs. 71/426	12 months
Ahmed, A	2006 USA	Open-label	Digoxin (n = 492)	Placebo (n = 496)	LVEF ≥ 45%, > 45 years age	66.7 ± 10.7 vs. 66.9 ± 9.9	21.5 vs. 22.6	115/492 vs. 116/496	81/492 vs. 81/496	61/492 vs. 73/496	89/492 vs. 108/496	37 months
Massie,BM	2008 USA	Single-blind	Irbesartan (n = 2067)	Placebo (n = 2061)	PRESERVE, LVEF ≥ 45%, > 60 years age	72 ± 7 vs. 72 ± 7	80 vs. 79	445/2067 vs. 436 /2061	311/2067 vs. 302 /2061	325/2067 vs. 336 /2061	291/2067 vs. 314 /2061	49.5 months
Yip, GW	2008 Hong Kong	Open-label	Irbesartan (n = 53) vs. Ramipril (n = 39)	Placebo (n = 47)	HK-PROBE, LVEF ≥ 45%, > 18 years age	75 ± 8.5 vs. 74 ± 6.1 vs. 73 ± 8.4	30.4 vs. 33.3 vs. 28.0	1/53 vs. 0/39 vs. 3/47	1/53 vs. 0/39 vs. 1/47	6/53 vs. 5/39 vs. 6/47	/	12 months
Solomon,SD	2012 USA	Double-blind	Sacubitril–valsartan (n = 149)	Valsartan (n = 152)	PARAMOUNT, LVEF ≥ 45%, > 18 years age	70.9 ± 9.4 vs. 71.2 ± 8.9	19 vs. 21	1/149 vs. 2/152	1/149 vs. 2/152	/	9/149 vs. 12/152	36 weeks
Edelmann, F	2013 Austria	Double-blind	Spironolactone (n = 213)	Placebo (n = 209)	Aldo-DHF, LVEF ≥ 50%, > 18 years age	67 ± 8 vs. 67 ± 8	15 vs. 12	1/213 vs. 0/209	1/213 vs. 0/2091	21/213 vs. 15/209	/	12 months
Yamamoto,K	2013 Japan	Open-label	Carvedilol (n = 120)	Placebo (n = 125)	DHF, LVEF > 40%, > 20 years age	73 ± 10 vs. 71 ± 11	15 vs. 8	18/120 vs. 21/1257	8/120 vs. 7/1255	21/120 vs. 27/125	25/120 vs. 31/125	24 months
Pitt, B	2014 USA	Double-blind	Spironolactone (n = 1722)	Placebo (n = 1723)	TOPCAT, LVEF ≥ 45%, > 18 years age	68.7 (61,76.4) vs. 68.7 (60.7,75.5)	33.4 vs. 32.6	252/1722 vs. 274/1723	160/1722 vs. 176/1723	206/1722 vs. 245/1723	/	3.3 years
Solomon, SD	2019 USA	Single-blind	Sacubitril–valsartan (n = 2407)	Valsartan (n = 2389)	PARAGON-HF, LVEF ≥ 45%, > 18 years age	72.7 ± 8.3 vs. 72.5 ± 8.5	19.3 vs. 20.3	342/2407 vs. 349/2389	204/2407 vs. 212/2389	690/2407 vs. 797/2389	202/2407 vs. 221/2389	4 years
Armstrong, PW	2020 Canada	Open-label	Vericiguat 15 mg (n = 264) vs. Vericiguat 10 mg (n = 262)	Placebo (n = 262)	VITALITY-HFpEF, LVEF ≥ 45%, > 45 years age	73.1 ± 9.1 vs. 72.2 ± 9.7 vs. 72.8 ± 9.4	42.4 vs. 41.4 vs. 40.5	10/264 vs. 15/262 vs. 7/262	8/264 vs. 12/262 vs. 4/262	/	/	24 weeks
Anker SD	2021 Germany	Double-blind	Empagliflozin (n = 2997)	Placebo (n = 2991)	EMPEROR-Presersed LVEF ≥ 50%	71.8 ± 9.3 vs. 71.9 ± 9.6		422 vs. 427	219 vs.244	259 vs 352	362 vs 485	36 months
Solomon, SD	2022 USA	Double-blind	Dapagliflflozin (n = 3131)	Placebo (n = 3132)	DELIVER LVEF > 40%	71.8 ± 9.6 vs. 71.5 ± 9.5	26.1 vs. 23.4	497 vs 526	231 vs. 261	329 vs 418	368 vs. 455	2.95 years

*HF* heart failure, *HFpEF* heart failure with preserved ejection fraction, *LVEF* left ventricular ejection fraction; “/” = no data available

### Primary and secondary endpoints

**Fig. 2:**
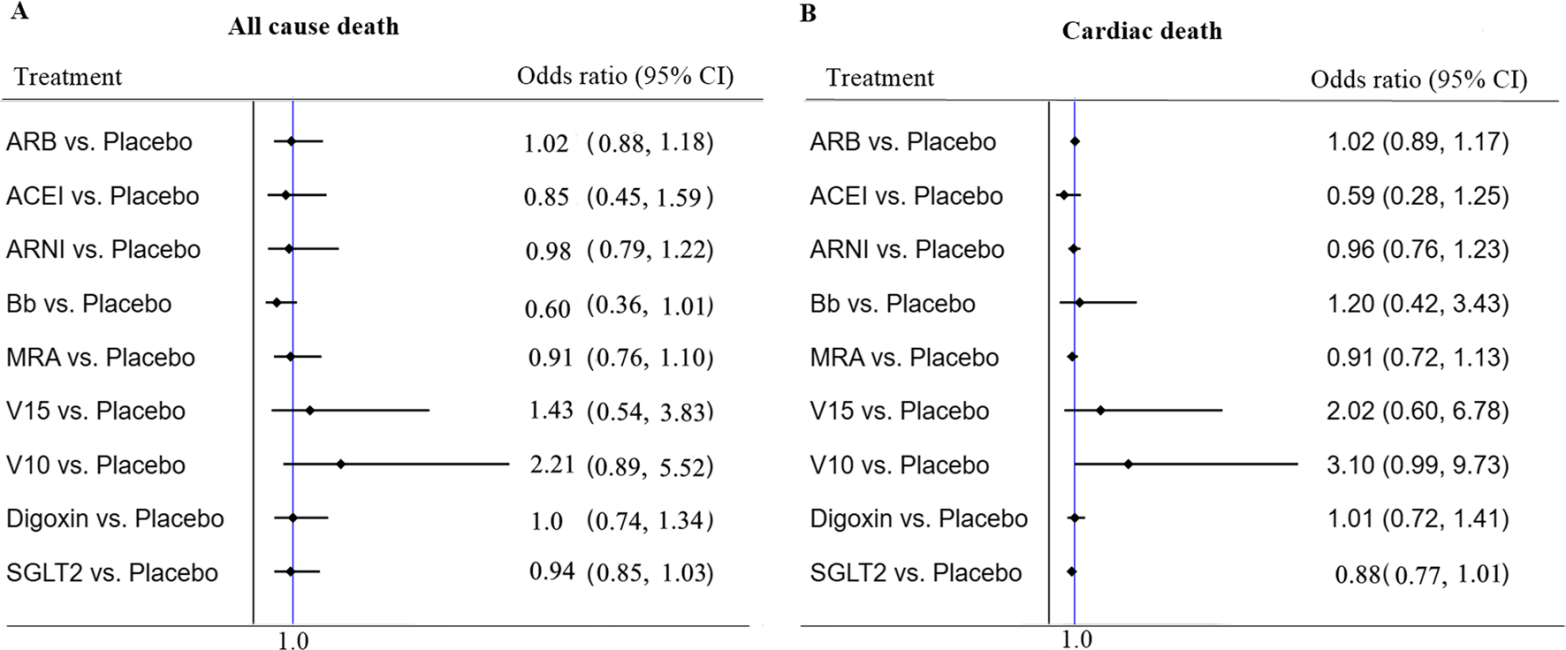
All-cause mortality (primary outcome): Forest plot (estimates as hazard ratio) of all trials. *Bb* beta blockers, *V15* vericiguat 15 mg, V10 vericiguat 10 mg

Thirteen of the 15 included RCTs (excluding Yusuf S et al.) reported all-cause death data. No drug treatments were found to significantly reduce all-cause death. Fourteen RCTs (excluding Wilbert S et al.) reported cardiac death data, whereas no difference was found in this network meta-analysis (Fig. 2).

**Fig. 3:**
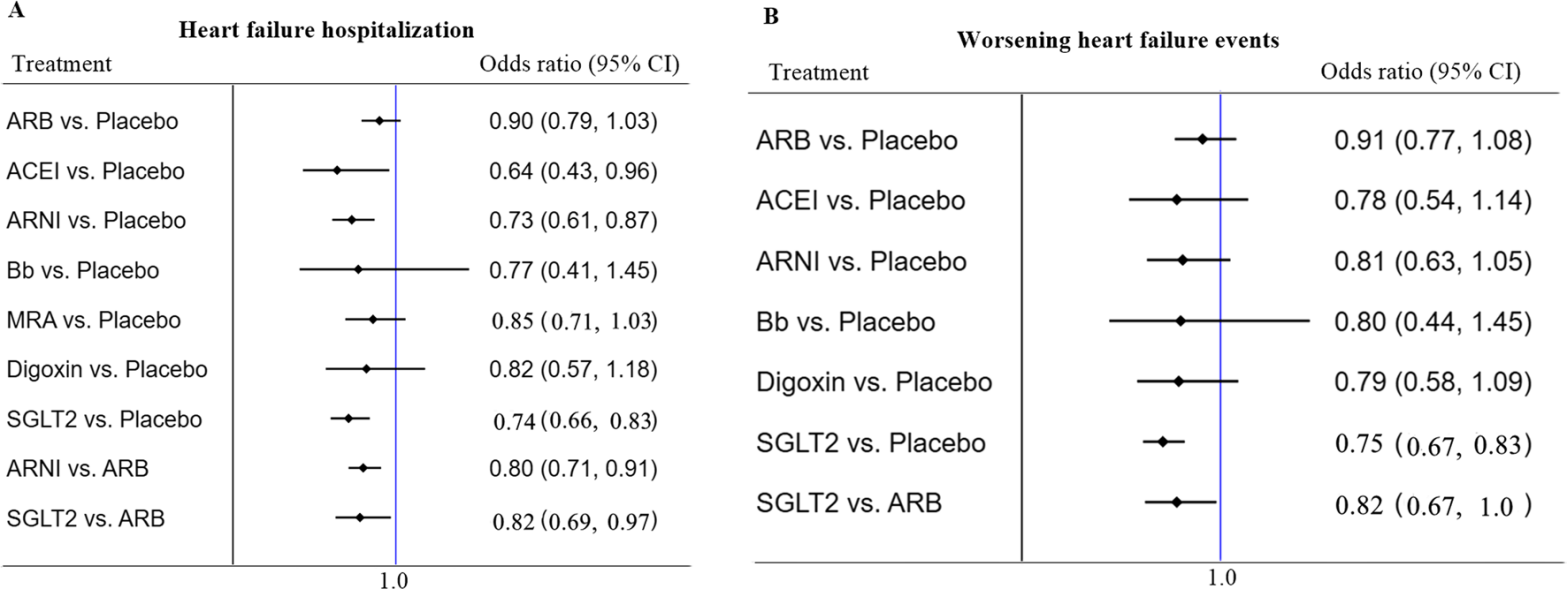
HF hospitalization and worsening HF events (secondary outcome): Forest plot (estimates as hazard ratio) of all trials

Compared to placebo, the ACEIs, ARNIs, and SGLT2 inhibitors significantly reduced hospitalization due to HF (HF hospitalization) [OR = 0.64 (95%CI 0.43 − 0.96), OR = 0.73 (95%CI 0.61 − 0.86), and OR = 0.74 (95%CI 0.66 − 0.83), respectively], without heterogeneity among studies. Only the SGLT2 inhibitors significantly reduced WHF events [OR = 0.75 (95%CI 0.67 − 0.83)] (Fig. 3).

The SUCRA rankogram plots for the primary endpoint showed that beta-blockers were the best treatment strategy for reducing all-cause death, followed by ACEIs, ARNIs, and SGLT2 (Additional file 2: Figure S1). However, no treatment was significantly different compared to placebo.

### Risk of bias assessment and publication bias

The Cochrane Collaboration tool was used for quality assessment. There were four studies with high attrition bias and selection bias, one with a large performance bias, and the remaining studies had low risk of bias (Additional file 2: Figure S2). No publication bias was found (Additional file 2: Figure S3). The CINeMA framework showed high quality of the included literature and low bias in the included studies (Additional file 3, 4, 5, 6).

## Discussion

The primary findings of this network meta-analysis are as follows: (i) There were no differences in all-cause and cardiac death between the drug treatments in patients with HFpEF; (ii) ARNIs, ACEIs, and SGLT2 inhibitors significantly reduced HF hospitalization, and only SGLT2 inhibitors reduced the risk of WHF events; and (iii) Angiotensin receptor blockers (ARBs) failed to reduce HF hospitalization.

HFpEF results from a complex interaction between risk factors, comorbidities, and cardiac pathology, affecting LV structure, hemodynamics, and systemic organ function. The underlying pathophysiological mechanisms of HFpEF include: (i) increased LV end-diastolic pressure (LVEDP) as evidenced by thickened LV walls and/or enlarged left atria [24, 25]; (ii) pulmonary vascular disease or dysfunction, and right ventricular failure [26]; and (iii) expansion of plasma volume and high plasma levels of natriuretic peptides [27–29]. These complex and diverse pathophysiological mechanisms make it difficult for the current treatment strategies to reduce mortality in HFpEF patients.

Our network meta-analysis showed that none of the current drug treatments have been able to reduce mortality in patients with HFpEF. Large RCTs focused on HFpEF outcomes, including PEP-CHF (perindopril) [13], CHARM-Preserved (candesartan) [11], I-PRESERVE (irbesartan) [15], TOPCAT (spironolactone) [20], DIG-Preserved (digoxin) [14], J-DHF (carvedilol) [19], VITALITY-HFpEF (vericiguat) [22], and PARAGON-HF (sacubitril/valsartan) [21], have failed to achieve their primary endpoints. However, more than 86%, 80%, and 24% of the included patients in the PARAGON-HF study received ACEI/ARB, beta-blockers, and MRA, respectively. The guideline-directed medical therapy (GDMT) may have overlapped with the mechanisms of ARNIs, resulting in no statistical difference in the primary endpoint. Additionally, subgroup analysis suggests a possible reduction (45–57%) in cardiac death in females. The EMPEROR-preserved study showed that empagliflozin significantly reduced the composite risk of cardiac death or HF hospitalization by 21% in patients with HFpEF compared to placebo, regardless of the presence of diabetes (HR 0.79, 95% CI 0.69–0.9, p < 0.0001) [23], while the DELIVER study showed that dapagliflozin significantly reduced the primary composite risk of worsening HF or cardiac death by 18% (HR 0.82, 95% CI 0.73–0.92, p < 0.001) [9]. However, it must be recognized that diabetes is a significant factor for HF hospitalization, and the cardiac mortality factor were not different between groups in the two SGLT2 inhibitor studies [OR = 0.88 (95%CI 0.73 − 1.07) and OR = 0.88 (95%CI 0.74 − 1.05), respectively]. These findings are consistent with a recent meta-analysis that included 12 RCTs (11 studies included subgroup analyses) comparing SGLT2 inhibitors with placebo in 10,883 patients with HFpEF [30].

In patients with HFpEF, most of whom are older, reducing the risk of HF hospitalization can also significantly improve the quality of life. Accordingly, ACEIs [EF ≥ 40%; OR = 0.64 (95%CI 0.43 − 0.96)], ARNIs [EF ≥ 40%; OR = 0.73 (95%CI 0.61 − 0.87)], and SGLT2 inhibitors [EF ≥ 50%; OR = 0.78 (95%CI 0.67 − 0.91) or EF ≥ 40%; OR = 0.74 (95%CI 0.66 − 0.83)] remain first choice treatments in terms of drug selection for HFpEF. There were no statistical between-group differences in drug interactions for the three drugs for reducing HF hospitalization. However, in the subgroup study of EMPEROR-preserved, SGLT2 inhibitors reduced HF hospitalization regarding of using ACEIs or ARNIs. Accordingly, ACEIs or ARNIs combined with SGLT2 inhibitors may further reduce endpoint events. The PEP-CHF study, which was a randomized double-blind trial, compared placebo with perindopril (4 mg/day) in patients aged 70 years with an EF ≥ 40% and found that perindopril reduced HF hospitalization [OR = 0.628 (95%CI 0.408 − 0.966)] and improved functional classes and the 6-min walk distance (6MWD) [13]. The 2019 PARAGON trial compared ARNI in 4822 patients with symptomatic HFpEF (EF ≥ 45%) and found no difference for reducing the risk of the primary composite outcome of HF hospitalization and cardiac death compared to valsartan [OR = 0.87 (95%CI 0.75–1.01)]. There was a reduction, although not significant, in HF hospitalization [OR = 0.85 (95%CI 0.72 − 1.00)] compared to ARBs in the PARAGON trial, whereas there was a significant reduction compared with placebo for the indirection comparison in our study.

In this meta-analysis, ARBs failed to reduce HF hospitalizations in HFpEF patients compared to placebo. This may be related to the fact that ACEIs are upstream blockers of the angiotensin-converting enzyme, and ARBs are downstream AT1 antagonists. ACEIs also preserve the kinin system, which may lead to the superiority of ACEIs over ARBs in anti-heart failure treatment. Secondly, ARNIs consist of ARBs and enkephalinase inhibitors that enhance the natriuretic peptide system by inhibiting enkephalinase. The PARAMOUNT study showed that ARNIs reduce NT-proBNP by approximately 23% compared to valsartan. Myocardial stiffness and myocardial fibrosis are the main physiological mechanisms in HFpEF [31]. Natriuretic peptides provide relief from breathlessness by rapidly reducing LVEDP and protecting the cardiovascular system by improving myocardial remodeling, including via its anti-hypertrophy anti-fibrotic effects [32]. Therefore, HFpEF patients who cannot tolerate ACEIs (dry cough) should perhaps be treated with ARNIs and not ARBs.

Only SGLT2 inhibitors reduced the risk of WHF events in patients with HFpEF [OR = 0.75 (95%CI 0.67 − 0.83)] following treatment with ACEIs, ARBs, or ARNIs at baseline. SGLT2 inhibitors have been shown to reduce the risk of HF hospitalization by 31% in patients with diabetes [33]. The dapagliflozin reduced the composite outcome of HF hospitalization and cardiac death in HFrEF by 26% in the DAPA-HF trial [34] and reduced the risk of WHF in HFpEF by 21% in the DELIVER study [9]. In the SOLOIST-WHF study, 1222 patients with type 2 diabetes mellitus who presented with WHF symptoms and were treated with intravenous diuretics were included in the study, of which 256 (21%) patients with LVEF ≥ 50% in the subgroup analysis, showing that sotagliflozin significantly reduced the risk of cardiac death and hospitalizations or emergency department visits due to WHF [HR = 0.78 (0.67–0.89)] [35]. Therefore, the 2022 AHA/ACC/HFSA guidelines for the management of heart failure firstly recommends SGLT2 inhibitors as new therapeutic agents in Class IIA, the strongest class into the treatment recommendations for HFpEF patients [36]. The mechanism of SGLT2 inhibitors for lowering the risk of HF hospitalization and adverse cardiovascular events may be explained as follows. (i) Cardiac stress-reducing effect: SGLT2 inhibitors promote the excretion of urinary sodium and glucose and thereby reduce cardiac preload. SGLT2 inhibitors can also block the renin–angiotensin–aldosterone system and reduce hypertension [37, 38]. (ii) Improve myocardial energy metabolism: SGLT2 inhibitors reduce oxidative stress and promote the breakdown of fatty acids into ketone bodies, thereby increasing ketone body levels in the body [39, 40]. This effect is distinct from ACEIs or ARNIs. Thus, the additional use of an SGLT2 inhibitor after ACEI or ARNI treatment may further reduce adverse events. (iii) Improving the pro-inflammatory properties of epicardial lipids (EAT) is an important target for therapeutic intervention in HFpEF. SGLT2i inhibitors can reduce EAT abnormalities, reduce water and sodium retention, and reduce the risk of HFpEF combined with atrial fibrillation. (iv) Anti-myocardial fibrosis and inhibition of ventricular remodeling: SGLT2 inhibitors can reduce the release of inflammatory factors, thereby reducing myocardial fibrosis [41, 42]. SGLT2 inhibitors can also reverse LV remodeling by reducing Na + and NHE-1 receptor activity in cardiomyocytes to slow myocardial fibrosis and cardiac hypertrophy [43].

Several limitations of this meta-analysis should be noted. First, HFpEF was defined as HF symptoms accompanied by an LVEF ≥ 50%. However, previous clinical studies have often included patients with an LVEF of 40–49%. A second limitation is that no study included the use of diuretics. Despite a lack of strong evidence, diuretics have been the first-line drug to relieve and alleviate HF symptoms due to fluid overload. Thus, the use of diuretics to reduce composite endpoints of HF hospitalization and WHF events should be considered in future RCTs. Third, the RELAX trial that included the use of a phosphodiesterase-5 inhibitor (sildenafil) did not provide any data on primary endpoints. Finally, this meta-analysis lacked data on 6MWD and KCCQ outcomes.

## Conclusion

No medications have been found to reduce the endpoint of mortality in HFpEF patients. ARNIs, ACEIs, or SGLT2 inhibitors significantly reduced the risk of HF hospitalization, but only SGLT2 inhibitors reduced WHF events.

## Supplementary Information

**Additional file 1:** **Table S1.** Final search strategy for PubMed. **Table 2**. Final search strategy for Clinical Trial gov of Controlled Trials. **Table 3**. Final search strategy for Cochrane Central Register of Controlled Trials

**Additional file 2:** **Figure 1.** Treatment strategy for all-cause mortality. **Figure 2**. Risk of bias in all trials. **Figure 3. **Assessment of risk of bias of the included studies (*I²*=0%)

**Additional file 3.** Heterogeneity of the network meta-analysis.

**Additional file 4.** Indirectness of the network meta-analysis.

**Additional file 5.** Incoherence of the network meta-analysis.

**Additional file 6.** Overal evaluattion of the network meta-analysis.

## Abbreviations

HFpEF: Heart failure with preserved ejection fraction
ACEI: Angiotensin-converting enzyme inhibitor
ARNI: Angiotensin receptor neprilysin inhibitor
SGLT2: Sodium-glucose cotransporter-2
MRA: Mineralocorticoid receptor antagonists
LVEDP: LV end-diastolic pressure
GDMT: Guideline-directed medical therapy
KCCQ: Kansas City Cardiomyopathy Questionnaire
6MWD: 6 Minutes walk distance
WHF: Worsening heart failure

## Declarations

### Availability of data and materials

The data that support the fndings of this study are available from the cor- responding author upon reasonable request.
