# Supplementary material for: Effect of pharmacological treatment on outcomes of heart failure with preserved ejection fraction: an updated systematic review and network meta-analysis of randomized controlled trials
Source: Cardiovasc Diabetol. 2022 Nov 8;21:237. doi: 10.1186/s12933-022-01679-2 (PMC9644566; doi:10.1186/s12933-022-01679-2)
Supplement: Supplementary file 1 — Additional file 1: Table S1. Final search strategy for PubMed. Table 2. Final search strategy for Clinical Trial gov of Controlled Trials. Table 3. Final search strategy for Cochrane Central Register of Controlled Trials [file 12933_2022_1679_MOESM1_ESM.doc]

eTable 1A. Final search strategy for PubMed

| #ID | Topic or intervention | Query | Records |
| --- | --- | --- | --- |
| #1 | Disease | (“heart failure with preserved ejection fraction”[Mesh]) OR (“diastolic heart failure”[Mesh] ) OR (“heart failure with preserved ejection fraction”[Title/Abstract] ) OR (“diastolic heart failure”[Title/Abstract]) | 5,338 |
| #2 | Study design | (“randomized controlled trial”[Publication Type] OR (randomized[Publication Typet]) OR “randomized controlled trial”[Title/Abstract] OR randomization [Title/Abstract] | 1,373,158 |
| #3 | angiotensin receptor neprilysin inhibitor | (“angiotensin receptor neprilysin inhibitor”[Mesh]) OR (“sacubitril–valsarta” [Mesh] ) OR (“LCZ696” [Mesh]) OR (“angiotensin receptor neprilysin inhibitor” [Title/Abstract]) OR (“sacubitril–valsarta”[Title/Abstract]) OR (“LCZ696” [Title/Abstract] ) | 666 |
| #4 | angiotensin converting enzyme inhibitors | (“angiotensin converting enzyme inhibitor”[Mesh]) OR (“perindopril” [Mesh] ) OR (“quinapril” [Mesh] ) OR (“perindopri” [Mesh] ) OR (“benazepril” [Mesh] ) OR (“catopril” [Mesh] ) OR (“fosinopril” [Mesh] ) OR (“enalapril” [Mesh] ) OR (“ramipril” [Mesh] ) OR (“angiotensin converting enzyme inhibitor” [Title/Abstract]) OR (“perindopril” [Title/Abstract]) OR (“quinapril” [Title/Abstract] ) OR (“perindopri” [Title/Abstract] ) OR (“benazepril” [Title/Abstract] ) OR (“catopril” [Title/Abstract] ) OR (“Fosinopril” [Title/Abstract] ) OR (“Enalapril” [Title/Abstract] ) OR (“ramipril” [Title/Abstract] ) | 21,408 |
| #5 | angiotensin receptor blockers | (“angiotensin receptor blockers”[Mesh]) OR (“irbesartan” [Mesh] ) OR (“valsartan” [Mesh] ) OR (“candesartan” [Mesh]) OR (“losartan” [Mesh]) OR (“angiotensin receptor blockers”[Title/Abstract]) OR (“Irbesartan” [Title/Abstract] ) OR (“valsartan” [Title/Abstract]) OR (“candesartan” [Title/Abstract]) OR (“losartan” [Title/Abstract] ) | 23,229 |
| #6 | beta blockers | (“beta blockers”[Mesh]) OR (“propranolol” [Mesh] ) OR (“carvedilol” [Mesh] ) OR (“metoprolol” [Mesh]) OR (“bisoprolol” [Mesh]) OR (“beta blockers”[Title/Abstract]) OR (“propranolol”[Title/Abstract]) OR (“carvedilol”[Title/Abstract]) OR (“metoprolol” [Title/Abstract] ) OR (“bisoprolol” [Title/Abstract] ) | 76,416 |
| #7 | mineralocorticoid receptor antagonists | (“mineralocorticoid receptor antagonists”[Mesh]) OR (“spirolactone” [Mesh] ) OR (“eplerenone” [Mesh] ) OR (“antisterone” [Mesh]) OR (“aldactone” [Mesh]) OR “mineralocorticoid receptor antagonists”[Title/Abstract]) OR (“spirolactone” [Title/Abstract]) OR (“eplerenone”[Title/Abstract]) OR (“antisterone” [Title/Abstract] ) OR (“aldactone” [Title/Abstract] ) | 7,091 |
| #8 | digoxin | (“digoxin”[Mesh]) OR (“digoxin” [Title/Abstract] ) | 16,791 |
| #9 | phosphodiesterase-5 inhibition or sidenafi | (“phosphodiesterase-5 inhibition”[Mesh]) OR (“sidenafi” [Mesh]) OR (“tadalafil” [Mesh]) OR (“phosphodiesterase-5 inhibition”[Title/Abstract]) OR (“sidenafi” [Title/Abstract]) OR (“tadalafil” [Title/Abstract] ) | 2,675 |
| #10 | sodium-glucose cotransporter-2 | (“sodium-glucose cotransporter-2”[Mesh]) OR (“dapagliflozin”[Mesh]) OR (“ canagliflozin” [Mesh] ) OR (“sodium-glucose cotransporter-2”[Title/Abstract]) OR (“dapagliflozin” [Title/Abstract] ) OR (“ canagliflozin” [Title/Abstract] ) | 5,445 |
| #11 | diuretic | (“loop diuretic ”[Mesh]) OR (“furosemide” [Mesh] ) OR (“bumetanide” [Mesh] ) OR (“torasemide” [Mesh]) OR (“azosemide” [Mesh]) OR (“eplerenone” [Mesh]) OR (“hydroclorotiazides” [Mesh]) OR (“loop diuretic ”[Title/Abstract]) OR (“furosemide”[Title/Abstract]) OR (“bumetanide”[Title/Abstract]) OR (“torasemide” [Title/Abstract]) OR (“azosemide” [Title/Abstract]) OR (“eplerenone” [Title/Abstract]) OR (“hydroclorotiazides” [Title/Abstract]) | 22,378 |
| #12 | Final query | #1 AND #2 AND (#3 OR #4 OR #5 OR #6 OR #7 OR #8 OR #9 OR #10 OR #11) | 452 |

**e**Table 2. Final search strategy for Clinical Trial gov of Controlled Trials

| #ID | Status | Query | Records |
| --- | --- | --- | --- |
| #1 | Completed | (heart failure with preserved ejection fraction OR diastolic heart failure) AND (angiotensin receptor neprilysin inhibitor OR angiotensin converting enzyme inhibitors OR angiotensin receptor blockers OR beta blockers OR mineralocorticoid receptor antagonists OR digoxin OR phosphodiesterase-5 inhibition or sidenafi OR sodium-glucose cotransporter-2 OR diuretic) | 12 |

**e**Table 3. Final search strategy for Cochrane Central Register of Controlled Trials

| #ID | Topic or intervention | Query | Records |
| --- | --- | --- | --- |
| #1 | Disease | heart failure with preserved ejection fraction OR diastolic heart failure | 5,557 |
| #2 | Study design | randomized controlled trial OR randomized | 1252587 |
| #3 | angiotensin receptor neprilysin inhibitor | angiotensin receptor neprilysin inhibitor OR sacubitril–valsarta OR LCZ696 | 243 |
| #4 | angiotensin converting enzyme inhibitors | angiotensin converting enzyme inhibitor OR perindopril OR quinapril OR perindopri OR benazepril OR catopril OR fosinopril OR enalapril OR ramipril | 9010 |
| #5 | angiotensin receptor blockers | angiotensin receptor blockers OR irbesartan OR valsartan OR candesartan OR losartan | 8597 |
| #6 | beta blockers | beta blocker OR propranolol OR carvedilol OR metoprolol OR bisoprolol | 13125 |
| #7 | mineralocorticoid receptor antagonists | mineralocorticoid receptor antagonists OR spirolactone OR eplerenone OR antisterone OR aldactone | 1293 |
| #8 | digoxin | digoxin | 1988 |
| #9 | phosphodiesterase-5 inhibition or sidenafi | phosphodiesterase-5 inhibition OR sidenafi OR tadalafil | 1239 |
| #10 | sodium-glucose cotransporter-2 | sodium-glucose cotransporter-2 OR dapagliflozin OR canagliflozin | 2749 |
| #11 | diuretic | loop diuretic OR furosemide OR bumetanide OR torasemide OR azosemide OR eplerenone OR hydroclorotiazides | 3882 |
| #12 | Final query | #1 AND #2 AND (#3 OR #4 OR #5 OR #6 OR #7 OR #8 OR #9 OR #10 OR #11) | 1244 |
