# Supplementary material for: Effect of pharmacological treatment on outcomes of heart failure with preserved ejection fraction: an updated systematic review and network meta-analysis of randomized controlled trials
Source: Cardiovasc Diabetol. 2022 Nov 8;21:237. doi: 10.1186/s12933-022-01679-2 (PMC9644566; doi:10.1186/s12933-022-01679-2)
Supplement: Supplementary file 2 — Additional file 2: Figure 1. Treatment strategy for all-cause mortality. Figure 2. Risk of bias in all trials. Figure 3. Assessment of risk of bias of the included studies (I²=0%) [file 12933_2022_1679_MOESM2_ESM.doc]

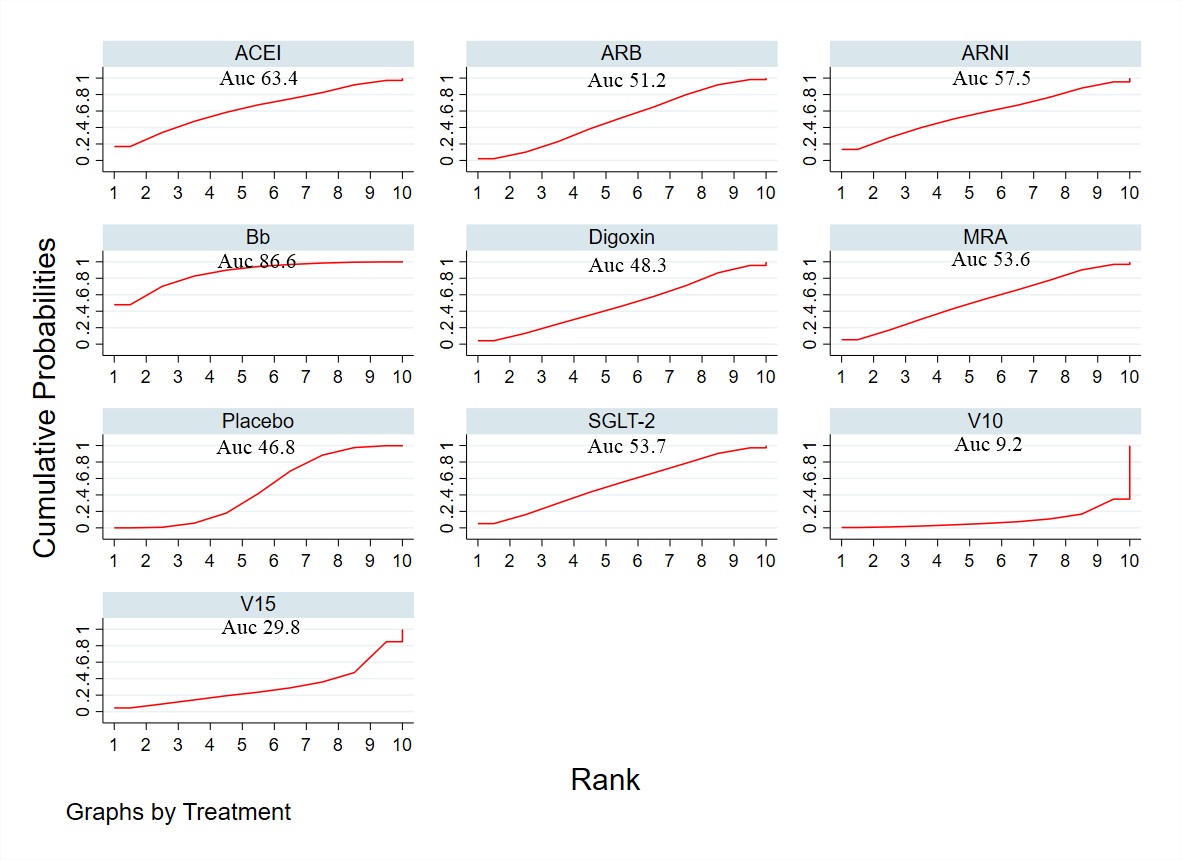


**eFigure 1. Treatment strategy for all-cause mortality**

SUCRA rankogram plots; ACEI, angiotensin-converting enzyme inhibitor; ARB, angiotensin receptor blocker; ARNI, angiotensin receptor neprilysin inhibitor; MRA, mineralocorticoid receptor antagonist; Bb, beta blockers; V15, vericiguat 15 mg; V10, vericiguat 10 mg.


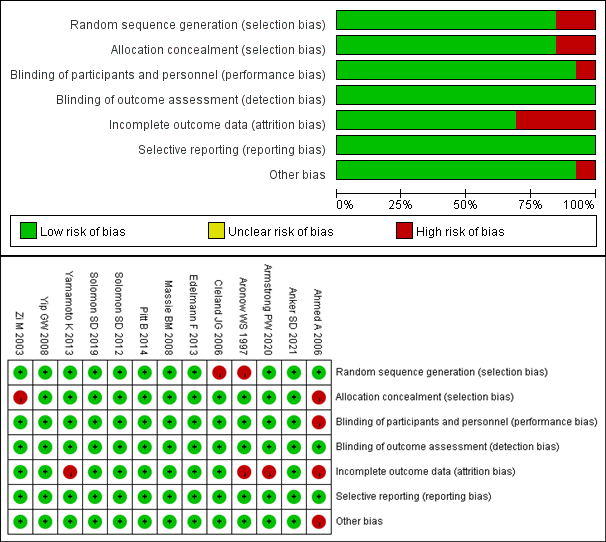


**eFigure 2. Risk of bias in all trials**


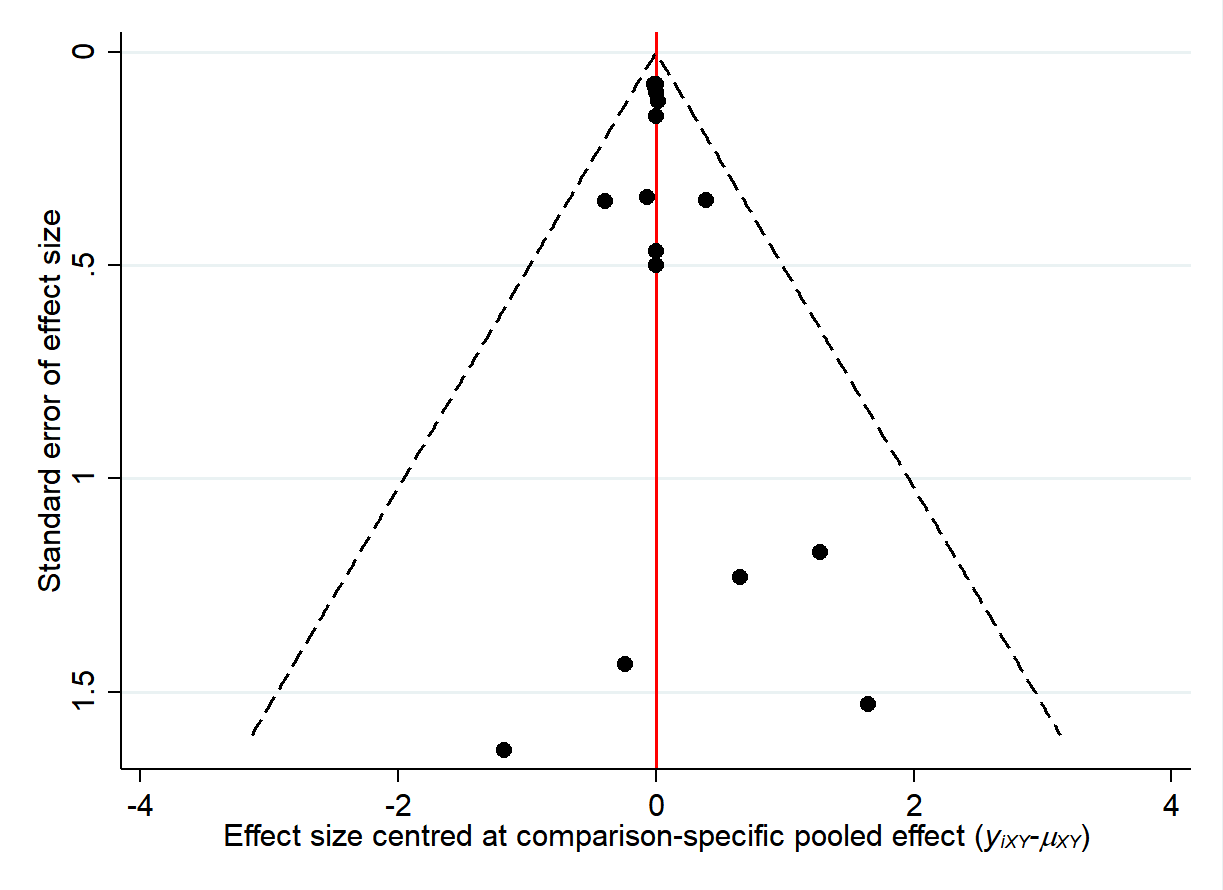


**eFigure 3. Assessment of risk of bias of the included studies (*I²*=0%)*.***
