# Supplementary material for: Effect of pharmacological treatment on outcomes of heart failure with preserved ejection fraction: an updated systematic review and network meta-analysis of randomized controlled trials
Source: Cardiovasc Diabetol. 2022 Nov 8;21:237. doi: 10.1186/s12933-022-01679-2 (PMC9644566; doi:10.1186/s12933-022-01679-2)
Supplement: Supplementary file 3 — Additional file 3. Heterogeneity of the network meta-analysis. [file 12933_2022_1679_MOESM3_ESM.pdf]

Confidence In Network Meta Analysis - CINeMA 2.0.0 - Project: **HFpEF network meta-analysis**

Heterogeneity

Define clinically important size of effect: Odds ratio 1

Relative effect estimates below **1.000** and above **1.000** are considered clinically important.

Importance of heterogeneity depends on the variability of effects in relation to a clinically important size of effect

The estimated value of between-study variance for the network meta-analysis is **0.000**

Comparison ACEI:ARB
Evidence: mixed

NMA estimate: **0.831**
95% intervals for NMA estimate
Confidence interval: **(0.433,1.594)**
Prediction interval: **(0.353,1.952)**

Confidence and prediction intervals **agree** in relation to clinically important effect

Heterogeneity judgment
No concerns

Comparison ACEI:Placebo
Evidence: mixed

NMA estimate: **0.845**
95% intervals for NMA estimate
Confidence interval: **(0.448,1.595)**
Prediction interval: **(0.368,1.944)**

Confidence and prediction intervals **agree** in relation to clinically important effect

Heterogeneity judgment
No concerns

Comparison ARB:ARNI
Evidence: mixed

NMA estimate: **1.036**
95% intervals for NMA estimate
Confidence interval: **(0.882,1.217)**
Prediction interval: **(0.839,1.279)**

Confidence and prediction intervals **agree** in relation to clinically important effect

Heterogeneity judgment
No concerns

Comparison ARB:Placebo
Evidence: mixed

NMA estimate: **1.018**
95% intervals for NMA estimate
Confidence interval: **(0.877,1.180)**
Prediction interval: **(0.837,1.236)**

Confidence and prediction intervals **agree** in relation to clinically important effect

Heterogeneity judgment
No concerns

Comparison Beta blockers:Placebo
Evidence: mixed

NMA estimate: **0.588**

95% intervals for NMA estimate

Confidence interval: **(0.363,0.953)**
Prediction interval: **(0.312,1.108)**

Prediction interval extends into clinically important effects in **both** directions

Heterogeneity judgment
Major concerns

Comparison Digoxin:Placebo
Evidence: mixed

NMA estimate: **0.999**

95% intervals for NMA estimate

Confidence interval: **(0.744,1.342)**
Prediction interval: **(0.679,1.471)**

Confidence and prediction intervals **agree** in relation to clinically important effect

Heterogeneity judgment
No concerns

Comparison MRA:Placebo
Evidence: mixed

NMA estimate: **0.910**

95% intervals for NMA estimate

Confidence interval: **(0.756,1.096)**
Prediction interval: **(0.714,1.161)**

Confidence and prediction intervals **agree** in relation to clinically important effect

Heterogeneity judgment
No concerns

Comparison Placebo:SGLT-2
Evidence: mixed

NMA estimate: **1.063**

95% intervals for NMA estimate

Confidence interval: **(0.918,1.232)**
Prediction interval: **(0.877,1.289)**

Confidence and prediction intervals **agree** in relation to clinically important effect

Heterogeneity judgment
No concerns

Comparison Placebo:Vericiguat 10mg
Evidence: mixed

NMA estimate: **0.452**

95% intervals for NMA estimate

Confidence interval: **(0.181,1.127)**
Prediction interval: **(0.136,1.499)**

Confidence and prediction intervals **agree** in relation to clinically important effect

Heterogeneity judgment
No concerns

Comparison Placebo:Vericiguat 15mg
Evidence: mixed

NMA estimate: **0.697**

95% intervals for NMA estimate

Confidence interval: **(0.261,1.860)**
Prediction interval: **(0.192,2.526)**

Confidence and prediction intervals **agree** in relation to clinically important effect

Heterogeneity judgment
No concerns

Comparison Vericiguat 10mg:Vericiguat 15mg
Evidence: mixed

NMA estimate: **1.542**

95% intervals for NMA estimate

Confidence interval: **(0.680,3.499)**
Prediction interval: **(0.527,4.516)**

Confidence and prediction intervals **agree** in relation to clinically important effect

Heterogeneity judgment
No concerns

Comparison ACEI:ARNI
Evidence: indirect

NMA estimate: **0.861**

95% intervals for NMA estimate

Confidence interval: **(0.440,1.684)**
Prediction interval: **(0.357,2.075)**

Confidence and prediction intervals **agree** in relation to clinically important effect

Heterogeneity judgment
No concerns

Comparison Evidence: ACEI:Beta blockers indirect

NMA estimate: **1.437**

95% intervals for NMA estimate

Confidence interval: **(0.647,3.193)**

Prediction interval: **(0.505,4.094)**

Confidence and prediction intervals **agree** in relation to clinically important effect

Heterogeneity judgment

No concerns

Comparison Evidence: indirect ACEI:Digoxin

NMA estimate: **0.846**

95% intervals for NMA estimate

Confidence interval: **(0.420,1.703)**

Prediction interval: **(0.338,2.118)**

Confidence and prediction intervals **agree** in relation to clinically important effect

Heterogeneity judgment

No concerns

Comparison Evidence: indirect ACEI:MRA

NMA estimate: **0.929**

95% intervals for NMA estimate

Confidence interval: **(0.479,1.799)**

Prediction interval: **(0.390,2.211)**

Confidence and prediction intervals **agree** in relation to clinically important effect

Heterogeneity judgment

No concerns

Comparison Evidence: indirect ACEI:SGLT-2

NMA estimate: **0.899**

95% intervals for NMA estimate

Confidence interval: **(0.468,1.725)**

Prediction interval: **(0.382,2.113)**

Confidence and prediction intervals **agree** in relation to clinically important effect

Heterogeneity judgment

No concerns

Comparison ACEI:Vericiguat 10mg
Evidence: indirect

NMA estimate: **0.382**

95% intervals for NMA estimate

Confidence interval: **(0.126,1.163)**
Prediction interval: **(0.089,1.645)**

Confidence and prediction intervals **agree** in relation to clinically important effect

Heterogeneity judgment
No concerns

Comparison ACEI:Vericiguat 15mg
Evidence: indirect

NMA estimate: **0.589**

95% intervals for NMA estimate

Confidence interval: **(0.183,1.897)**
Prediction interval: **(0.127,2.730)**

Confidence and prediction intervals **agree** in relation to clinically important effect

Heterogeneity judgment
No concerns

Comparison ARB:Beta blockers
Evidence: indirect

NMA estimate: **1.731**

95% intervals for NMA estimate

Confidence interval: **(1.044,2.869)**
Prediction interval: **(0.892,3.359)**

Prediction interval extends into clinically important effects in **both** directions

Heterogeneity judgment
Major concerns

Comparison ARB:Digoxin
Evidence: indirect

NMA estimate: **1.018**

95% intervals for NMA estimate

Confidence interval: **(0.732,1.416)**
Prediction interval: **(0.661,1.570)**

Confidence and prediction intervals **agree** in relation to clinically important effect

Heterogeneity judgment
No concerns

Comparison ARB:MRA
Evidence: indirect

NMA estimate: **1.118**

95% intervals for NMA estimate

Confidence interval: **(0.882,1.418)**
Prediction interval: **(0.819,1.527)**

Confidence and prediction intervals **agree** in relation to clinically important effect

Heterogeneity judgment
No concerns

Comparison ARB:SGLT-2
Evidence: indirect

NMA estimate: **1.082**

95% intervals for NMA estimate

Confidence interval: **(0.878,1.333)**
Prediction interval: **(0.823,1.423)**

Confidence and prediction intervals **agree** in relation to clinically important effect

Heterogeneity judgment
No concerns

Comparison ARB:Vericiguat 10mg
Evidence: indirect

NMA estimate: **0.460**

95% intervals for NMA estimate

Confidence interval: **(0.182,1.161)**
Prediction interval: **(0.137,1.549)**

Confidence and prediction intervals **agree** in relation to clinically important effect

Heterogeneity judgment
No concerns

Comparison ARB:Vericiguat 15mg
Evidence: indirect

NMA estimate: **0.709**

95% intervals for NMA estimate

Confidence interval: **(0.263,1.914)**
Prediction interval: **(0.193,2.608)**

Confidence and prediction intervals **agree** in relation to clinically important effect

Heterogeneity judgment
No concerns

Comparison Evidence: indirect **ARNI:Beta blockers**

NMA estimate: **1.670**

95% intervals for NMA estimate

Confidence interval: **(0.983,2.840)**

Prediction interval: **(0.833,3.350)**

Confidence and prediction intervals **agree** in relation to clinically important effect

Heterogeneity judgment

No concerns

Comparison Evidence: indirect **ARNI:Digoxin**

NMA estimate: **0.983**

95% intervals for NMA estimate

Confidence interval: **(0.681,1.419)**

Prediction interval: **(0.607,1.591)**

Confidence and prediction intervals **agree** in relation to clinically important effect

Heterogeneity judgment

No concerns

Comparison Evidence: indirect **ARNI:MRA**

NMA estimate: **1.079**

95% intervals for NMA estimate

Confidence interval: **(0.810,1.438)**

Prediction interval: **(0.741,1.572)**

Confidence and prediction intervals **agree** in relation to clinically important effect

Heterogeneity judgment

No concerns

Comparison Evidence: indirect **ARNI:Placebo**

NMA estimate: **0.982**

95% intervals for NMA estimate

Confidence interval: **(0.789,1.223)**

Prediction interval: **(0.737,1.309)**

Confidence and prediction intervals **agree** in relation to clinically important effect

Heterogeneity judgment

No concerns

Comparison ARNI:SGLT-2
Evidence: indirect

NMA estimate: **1.044**

95% intervals for NMA estimate

Confidence interval: **(0.802,1.359)**
Prediction interval: **(0.739,1.476)**

Confidence and prediction intervals **agree** in relation to clinically important effect

Heterogeneity judgment
No concerns

Comparison ARNI:Vericiguat 10mg
Evidence: indirect

NMA estimate: **0.444**

95% intervals for NMA estimate

Confidence interval: **(0.173,1.136)**
Prediction interval: **(0.129,1.523)**

Confidence and prediction intervals **agree** in relation to clinically important effect

Heterogeneity judgment
No concerns

Comparison ARNI:Vericiguat 15mg
Evidence: indirect

NMA estimate: **0.685**

95% intervals for NMA estimate

Confidence interval: **(0.251,1.872)**
Prediction interval: **(0.183,2.560)**

Confidence and prediction intervals **agree** in relation to clinically important effect

Heterogeneity judgment
No concerns

Comparison Beta blockers:Digoxin
Evidence: indirect

NMA estimate: **0.588**

95% intervals for NMA estimate

Confidence interval: **(0.334,1.036)**
Prediction interval: **(0.280,1.236)**

Confidence and prediction intervals **agree** in relation to clinically important effect

Heterogeneity judgment
No concerns

Comparison
Evidence: Beta blockers:MRA indirect

NMA estimate: **0.646**

95% intervals for NMA estimate

Confidence interval: **(0.385,1.084)**
Prediction interval: **(0.328,1.274)**

Confidence and prediction intervals **agree** in relation to clinically important effect

Heterogeneity judgment
No concerns

Comparison
Beta blockers:SGLT-2
Evidence: indirect

NMA estimate: **0.625**

95% intervals for NMA estimate

Confidence interval: **(0.377,1.036)**
Prediction interval: **(0.322,1.213)**

Confidence and prediction intervals **agree** in relation to clinically important effect

Heterogeneity judgment
No concerns

Comparison
Beta blockers:Vericiguat 10mg
Evidence: indirect

NMA estimate: **0.266**

95% intervals for NMA estimate

Confidence interval: **(0.095,0.747)**
Prediction interval: **(0.068,1.031)**

Prediction interval extends into clinically important effects in **both** directions

Heterogeneity judgment
Major concerns

Comparison
Beta blockers:Vericiguat 15mg
Evidence: indirect

NMA estimate: **0.410**

95% intervals for NMA estimate

Confidence interval: **(0.137,1.224)**
Prediction interval: **(0.098,1.721)**

Confidence and prediction intervals **agree** in relation to clinically important effect

Heterogeneity judgment
No concerns

Comparison Digoxin:MRA
Evidence: indirect

NMA estimate: **1.098**

95% intervals for NMA estimate

Confidence interval: **(0.775,1.555)**
Prediction interval: **(0.695,1.733)**

Confidence and prediction intervals **agree** in relation to clinically important effect

Heterogeneity judgment
No concerns

Comparison Digoxin:SGLT-2
Evidence: indirect

NMA estimate: **1.062**

95% intervals for NMA estimate

Confidence interval: **(0.764,1.477)**
Prediction interval: **(0.690,1.636)**

Confidence and prediction intervals **agree** in relation to clinically important effect

Heterogeneity judgment
No concerns

Comparison Digoxin:Vericiguat 10mg
Evidence: indirect

NMA estimate: **0.452**

95% intervals for NMA estimate

Confidence interval: **(0.173,1.180)**
Prediction interval: **(0.128,1.592)**

Confidence and prediction intervals **agree** in relation to clinically important effect

Heterogeneity judgment
No concerns

Comparison Digoxin:Vericiguat 15mg
Evidence: indirect

NMA estimate: **0.697**

95% intervals for NMA estimate

Confidence interval: **(0.250,1.941)**
Prediction interval: **(0.182,2.671)**

Confidence and prediction intervals **agree** in relation to clinically important effect

Heterogeneity judgment
No concerns

Comparison MRA:SGLT-2
Evidence: indirect

NMA estimate: **0.968**
95% intervals for NMA estimate
Confidence interval: **(0.764,1.226)**
Prediction interval: **(0.709,1.320)**

Confidence and prediction intervals **agree** in relation to clinically important effect

Heterogeneity judgment
No concerns

Comparison MRA:Vericiguat 10mg
Evidence: indirect

NMA estimate: **0.411**
95% intervals for NMA estimate
Confidence interval: **(0.162,1.046)**
Prediction interval: **(0.121,1.398)**

Confidence and prediction intervals **agree** in relation to clinically important effect

Heterogeneity judgment
No concerns

Comparison MRA:Vericiguat 15mg
Evidence: indirect

NMA estimate: **0.635**
95% intervals for NMA estimate
Confidence interval: **(0.234,1.723)**
Prediction interval: **(0.171,2.352)**

Confidence and prediction intervals **agree** in relation to clinically important effect

Heterogeneity judgment
No concerns

Comparison SGLT-2:Vericiguat 10mg
Evidence: indirect

NMA estimate: **0.425**
95% intervals for NMA estimate
Confidence interval: **(0.168,1.073)**
Prediction interval: **(0.126,1.432)**

Confidence and prediction intervals **agree** in relation to clinically important effect

Heterogeneity judgment
No concerns

Comparison
SGLT-2:Vericiguat 15mg
Evidence: indirect

NMA estimate: **0.656**

95% intervals for NMA estimate

Confidence interval: **(0.243,1.769)**
Prediction interval: **(0.178,2.410)**

Confidence and prediction intervals **agree** in relation to clinically important effect

Heterogeneity judgment
No concerns
