# Supplementary material for: Effect of pharmacological treatment on outcomes of heart failure with preserved ejection fraction: an updated systematic review and network meta-analysis of randomized controlled trials
Source: Cardiovasc Diabetol. 2022 Nov 8;21:237. doi: 10.1186/s12933-022-01679-2 (PMC9644566; doi:10.1186/s12933-022-01679-2)
Supplement: Supplementary file 4 — Additional file 4. Indirectness of the network meta-analysis. [file 12933_2022_1679_MOESM4_ESM.pdf]

Confidence In Network Meta Analysis - CINeMA 2.0.0 - Project: **HFpEF network meta-analysis**

Indirectness

13 total studies
11 : low **2** : moderate

Indirectness contributions

The bar chart shows the contributions of each study to the network estimate

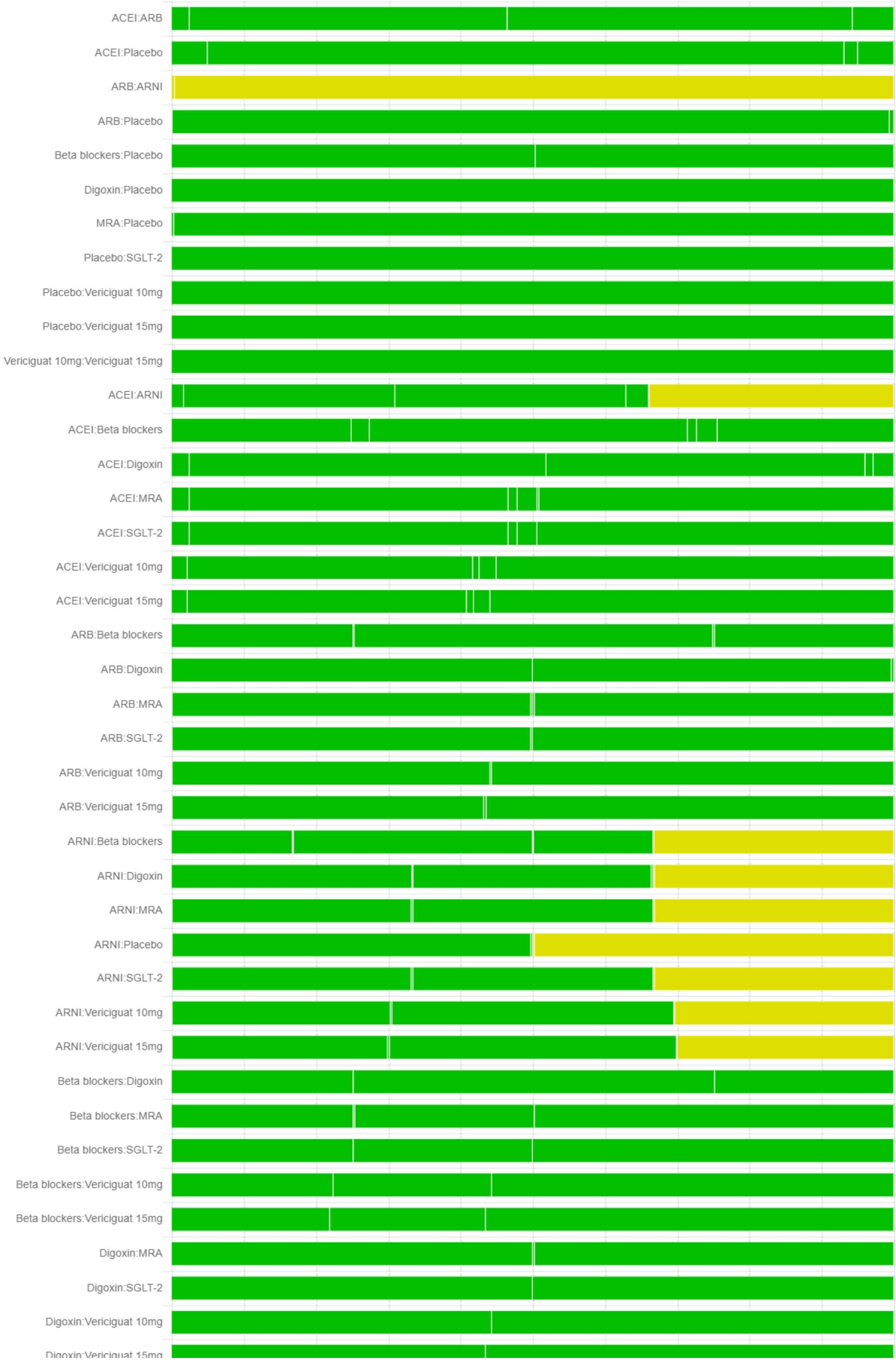
ACEI:ARB
ACEI:Placebo
ARB:ARNI
ARB:Placebo
Beta blockers:Placebo
Digoxin:Placebo
MRA:Placebo
Placebo:SGLT-2
Placebo:Vericiguat 10mg
Placebo:Vericiguat 15mg
Vericiguat 10mg:Vericiguat 15mg
ACEI:ARNI
ACEI:Beta blockers
ACEI:Digoxin
ACEI:MRA
ACEI:SGLT-2
ACEI:Vericiguat 10mg
ACEI:Vericiguat 15mg
ARB:Beta blockers
ARB:Digoxin
ARB:MRA
ARB:SGLT-2
ARB:Vericiguat 10mg
ARB:Vericiguat 15mg
ARNI:Beta blockers
ARNI:Digoxin
ARNI:MRA
ARNI:Placebo
ARNI:SGLT-2
ARNI:Vericiguat 10mg
ARNI:Vericiguat 15mg
Beta blockers:Digoxin
Beta blockers:MRA
Beta blockers:SGLT-2
Beta blockers:Vericiguat 10mg
Beta blockers:Vericiguat 15mg
Digoxin:MRA
Digoxin:SGLT-2
Digoxin:Vericiguat 10mg
Digoxin:Vericiguat 15mg

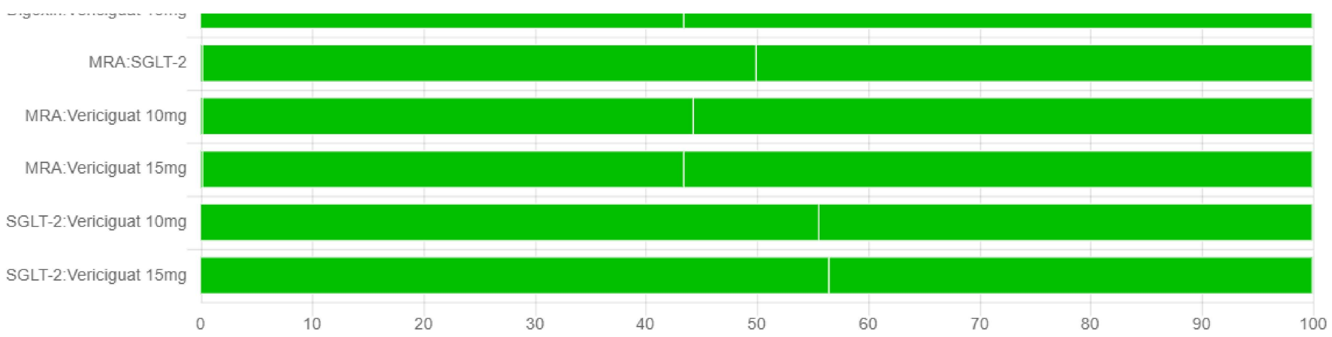

Selected rule: Average

2 comparisons were manually updated

Comparison ACEI:ARB
Evidence: mixed

Majority: **No concerns**
Average: **No concerns**
Highest: **Some concerns**

NMA judgment
No concerns

Comparison ACEI:Placebo
Evidence: mixed

Majority: **No concerns**
Average: **No concerns**
Highest: **Some concerns**

NMA judgment
No concerns

Comparison ARB:ARNI
Evidence: mixed

Majority: **Some concerns**
Average: **Some concerns**
Highest: **Some concerns**

NMA judgment
Some concerns

Comparison ARB:Placebo
Evidence: mixed

Majority: **No concerns**
Average: **No concerns**
Highest: **Some concerns**

NMA judgment
No concerns

Comparison Beta blockers:Placebo
Evidence: mixed

Majority: **No concerns**
Average: **No concerns**
Highest: **Some concerns**

NMA judgment
No concerns

Comparison Digoxin:Placebo
Evidence: mixed

Majority: **No concerns**
Average: **No concerns**
Highest: **Some concerns**

NMA judgment
No concerns

Comparison MRA:Placebo
Evidence: mixed

Majority: **No concerns**
Average: **No concerns**
Highest: **Some concerns**

NMA judgment
No concerns

Comparison Placebo:SGLT-2
Evidence: mixed

Majority: **No concerns**
Average: **No concerns**
Highest: **Some concerns**

NMA judgment
Major concerns

Comparison Placebo:Vericiguat 10mg
Evidence: mixed

Majority: **No concerns**
Average: **No concerns**
Highest: **Some concerns**

NMA judgment
No concerns

Comparison Placebo:Vericiguat 15mg
Evidence: mixed

Majority: **No concerns**
Average: **No concerns**
Highest: **Some concerns**

NMA judgment
No concerns

Comparison Vericiguat 10mg:Vericiguat 15mg
Evidence: mixed

Majority: **No concerns**
Average: **No concerns**
Highest: **Some concerns**

NMA judgment
No concerns

Comparison ACEI:ARNI
Evidence: indirect

Majority: **No concerns**
Average: **No concerns**
Highest: **Some concerns**

NMA judgment
Major concerns

Comparison ACEI:Beta blockers
Evidence: indirect

Majority: **No concerns**
Average: **No concerns**
Highest: **Some concerns**

NMA judgment
No concerns

Comparison ACEI:Digoxin
Evidence: indirect

Majority: **No concerns**
Average: **No concerns**
Highest: **Some concerns**

NMA judgment
No concerns

Comparison ACEI:MRA
Evidence: indirect

Majority: **No concerns**
Average: **No concerns**
Highest: **Some concerns**

NMA judgment
No concerns

Comparison ACEI:SGLT-2
Evidence: indirect

Majority: **No concerns**
Average: **No concerns**
Highest: **Some concerns**

NMA judgment
No concerns

Comparison ACEI:Vericiguat 10mg
Evidence: indirect

Majority: **No concerns**
Average: **No concerns**
Highest: **Some concerns**

NMA judgment
No concerns

Comparison ACEI:Vericiguat 15mg
Evidence: indirect

Majority: **No concerns**
Average: **No concerns**
Highest: **Some concerns**

NMA judgment
No concerns

Comparison ARB:Beta blockers
Evidence: indirect

Majority: **No concerns**
Average: **No concerns**
Highest: **Some concerns**

NMA judgment
No concerns

Comparison ARB:Digoxin
Evidence: indirect

Majority: **No concerns**
Average: **No concerns**
Highest: **Some concerns**

NMA judgment
No concerns

Comparison ARB:MRA
Evidence: indirect

Majority: **No concerns**
Average: **No concerns**
Highest: **Some concerns**

NMA judgment
No concerns

Comparison ARB:SGLT-2
Evidence: indirect

Majority: **No concerns**
Average: **No concerns**
Highest: **Some concerns**

NMA judgment
No concerns

Comparison ARB:Vericiguat 10mg
Evidence: indirect

Majority: **No concerns**
Average: **No concerns**
Highest: **Some concerns**

NMA judgment
No concerns

Comparison ARB:Vericiguat 15mg
Evidence: indirect

Majority: **No concerns**
Average: **No concerns**
Highest: **Some concerns**

NMA judgment
No concerns

Comparison ARNI:Beta blockers
Evidence: indirect

Majority: **No concerns**
Average: **No concerns**
Highest: **Some concerns**

NMA judgment
No concerns

Comparison ARNI:Digoxin
Evidence: indirect

Majority: **No concerns**
Average: **No concerns**
Highest: **Some concerns**

NMA judgment
No concerns

Comparison ARNI:MRA
Evidence: indirect

Majority: **No concerns**
Average: **No concerns**
Highest: **Some concerns**

NMA judgment
No concerns

Comparison ARNI:Placebo
Evidence: indirect

Majority: **No concerns**
Average: **No concerns**
Highest: **Some concerns**

NMA judgment
No concerns

Comparison ARNI:SGLT-2
Evidence: indirect

Majority: **No concerns**
Average: **No concerns**
Highest: **Some concerns**

NMA judgment
No concerns

Comparison ARNI:Vericiguat 10mg
Evidence: indirect

Majority: **No concerns**
Average: **No concerns**
Highest: **Some concerns**

NMA judgment
No concerns

Comparison ARNI:Vericiguat 15mg
Evidence: indirect

Majority: **No concerns**
Average: **No concerns**
Highest: **Some concerns**

NMA judgment
No concerns

Comparison Beta blockers:Digoxin
Evidence: indirect

Majority: **No concerns**
Average: **No concerns**
Highest: **Some concerns**

NMA judgment
No concerns

Comparison Beta blockers:MRA
Evidence: indirect

Majority: **No concerns**
Average: **No concerns**
Highest: **Some concerns**

NMA judgment
No concerns

Comparison Beta blockers:SGLT-2
Evidence: indirect

Majority: **No concerns**
Average: **No concerns**
Highest: **Some concerns**

NMA judgment
No concerns

Comparison Beta blockers:Vericiguat 10mg
Evidence: indirect

Majority: **No concerns**
Average: **No concerns**
Highest: **Some concerns**

NMA judgment
No concerns

Comparison Beta blockers:Vericiguat 15mg
Evidence: indirect

Majority: **No concerns**
Average: **No concerns**
Highest: **Some concerns**

NMA judgment
No concerns

Comparison Digoxin:MRA
Evidence: indirect

Majority: **No concerns**
Average: **No concerns**
Highest: **Some concerns**

NMA judgment
No concerns

Comparison Digoxin:SGLT-2
Evidence: indirect

Majority: **No concerns**
Average: **No concerns**
Highest: **Some concerns**

NMA judgment
No concerns

Comparison Digoxin:Vericiguat 10mg
Evidence: indirect

Majority: **No concerns**
Average: **No concerns**
Highest: **Some concerns**

NMA judgment
No concerns

Comparison Digoxin:Vericiguat 15mg
Evidence: indirect

Majority: **No concerns**
Average: **No concerns**
Highest: **Some concerns**

NMA judgment
No concerns

Comparison MRA:SGLT-2
Evidence: indirect

Majority: **No concerns**
Average: **No concerns**
Highest: **Some concerns**

NMA judgment
No concerns

Comparison MRA:Vericiguat 10mg
Evidence: indirect

Majority: **No concerns**
Average: **No concerns**
Highest: **Some concerns**

NMA judgment
No concerns

Comparison MRA:Vericiguat 15mg
Evidence: indirect

Majority: **No concerns**
Average: **No concerns**
Highest: **Some concerns**

NMA judgment
No concerns

Comparison SGLT-2:Vericiguat 10mg
Evidence: indirect

Majority: **No concerns**
Average: **No concerns**
Highest: **Some concerns**

NMA judgment
No concerns

Comparison SGLT-2:Vericiguat 15mg
Evidence: indirect

Majority: **No concerns**
Average: **No concerns**
Highest: **Some concerns**

NMA judgment
No concerns
