# Supplementary material for: Effect of pharmacological treatment on outcomes of heart failure with preserved ejection fraction: an updated systematic review and network meta-analysis of randomized controlled trials
Source: Cardiovasc Diabetol. 2022 Nov 8;21:237. doi: 10.1186/s12933-022-01679-2 (PMC9644566; doi:10.1186/s12933-022-01679-2)
Supplement: Supplementary file 5 — Additional file 5. Incoherence of the network meta-analysis. [file 12933_2022_1679_MOESM5_ESM.pdf]

Confidence In Network Meta Analysis - CINeMA 2.0.0 - Project: **HFpEF network meta-analysis**

Incoherence

Define clinically important size of effect: Odds ratio 1

Relative effect estimates below **1.000** and above **1.000** are considered clinically important.

Importance of Incoherence depends on the variability of direct and indirect effects in relation to a clinically important size of effect

Global test based on a random-effects design-by-treatment interaction model

χ^2 statistic: 1.912 (2 degrees of freedom), P value: 0.384

Local tests: Separating indirect from direct evidence

**Comparison**	**ACEI:ARB**
**Evidence: mixed**	
NMA odds ratio:	**0.831(0.433,1.594)**
Direct odds ratio:	**0.443(0.018,11.167)**
Indirect odds ratio:	**0.853(0.439,1.659)**
**Inconsistency measures**	
Ratio of odds ratios:	**0.519(0.019,14.009)**
P value:	**0.697**
Incoherence judgment	Some concerns

**Comparison**	**ACEI:Placebo**
**Evidence: mixed**	
NMA odds ratio:	**0.845(0.448,1.595)**
Direct odds ratio:	**0.835(0.442,1.577)**
Indirect odds ratio:	**407.198(0.000,834213564.430)**
**Inconsistency measures**	
Ratio of odds ratios:	**0.002(0.000,4261.174)**
P value:	**0.404**
Incoherence judgment	Some concerns

**Comparison**	**ARB:ARNI**
**Evidence: direct**	
Direct odds ratio:	**1.036(0.882,1.217)**
**Inconsistency measures:** Not applicable	
Incoherence judgment	No concerns

Comparison **ARB:Placebo**
Evidence: direct
Direct odds ratio: **1.016(0.876,1.179)**
Inconsistency measures: Not applicable
Incoherence judgment: No concerns

Comparison **Beta blockers:Placebo**
Evidence: direct
Direct odds ratio: **0.588(0.363,0.953)**
Inconsistency measures: Not applicable
Incoherence judgment: No concerns

Comparison **Digoxin:Placebo**
Evidence: direct
Direct odds ratio: **0.999(0.744,1.342)**
Inconsistency measures: Not applicable
Incoherence judgment: Major concerns

Comparison **MRA:Placebo**
Evidence: direct
Direct odds ratio: **0.910(0.756,1.096)**
Inconsistency measures: Not applicable
Incoherence judgment: No concerns

Comparison **Placebo:SGLT-2**
Evidence: direct
Direct odds ratio: **1.063(0.918,1.232)**
Inconsistency measures: Not applicable
Incoherence judgment: No concerns

Comparison **Placebo:Vericiguat 10mg**
Evidence: direct
Direct odds ratio: **0.452(0.181,1.127)**
Inconsistency measures: Not applicable
Incoherence judgment: No concerns

Comparison **Placebo:Vericiguat 15mg**
Evidence: direct
Direct odds ratio: **0.697(0.261,1.860)**
Inconsistency measures: Not applicable
Incoherence judgment: No concerns

Comparison **Vericiguat 10mg:Vericiguat 15mg**
Evidence: direct
Direct odds ratio: **1.542(0.680,3.499)**
Inconsistency measures: Not applicable
Incoherence judgment: No concerns

Comparison **ACEI:ARNI**
Evidence: indirect
Indirect odds ratio: **0.861(0.440,1.684)**
Inconsistency measures: Not applicable
Incoherence judgment No concerns

Comparison **ACEI:Beta blockers**
Evidence: indirect
Indirect odds ratio: **1.437(0.647,3.193)**
Inconsistency measures: Not applicable
Incoherence judgment Some concerns

Comparison **ACEI:Digoxin**
Evidence: indirect
Indirect odds ratio: **0.846(0.420,1.703)**
Inconsistency measures: Not applicable
Incoherence judgment No concerns

Comparison **ACEI:MRA**
Evidence: indirect
Indirect odds ratio: **0.929(0.479,1.799)**
Inconsistency measures: Not applicable
Incoherence judgment No concerns

Comparison **ACEI:SGLT-2**
Evidence: indirect
Indirect odds ratio: **0.899(0.468,1.725)**
Inconsistency measures: Not applicable
Incoherence judgment No concerns

Comparison **ACEI:Vericiguat 10mg**
Evidence: indirect
Indirect odds ratio: **0.382(0.126,1.163)**
Inconsistency measures: Not applicable
Incoherence judgment No concerns

Comparison **ACEI:Vericiguat 15mg**
Evidence: indirect
Indirect odds ratio: **0.589(0.183,1.897)**
Inconsistency measures: Not applicable
Incoherence judgment No concerns

Comparison **ARB:Beta blockers**
Evidence: indirect
Indirect odds ratio: **1.731(1.044,2.869)**
Inconsistency measures: Not applicable
Incoherence judgment No concerns

Comparison **ARB:Digoxin**
Evidence: indirect
Indirect odds ratio: **1.018(0.732,1.416)**
Inconsistency measures: Not applicable
Incoherence judgment No concerns

Comparison **ARB:MRA**
Evidence: indirect
Indirect odds ratio: **1.118(0.882,1.418)**
Inconsistency measures: Not applicable
Incoherence judgment No concerns

Comparison **ARB:SGLT-2**
Evidence: indirect
Indirect odds ratio: **1.082(0.878,1.333)**
Inconsistency measures: Not applicable
Incoherence judgment No concerns

Comparison **ARB:Vericiguat 10mg**
Evidence: indirect
Indirect odds ratio: **0.460(0.182,1.161)**
Inconsistency measures: Not applicable
Incoherence judgment No concerns

Comparison **ARB:Vericiguat 15mg**
Evidence: indirect
Indirect odds ratio: **0.709(0.263,1.914)**
Inconsistency measures: Not applicable
Incoherence judgment No concerns

Comparison **ARNI:Beta blockers**
Evidence: indirect
Indirect odds ratio: **1.670(0.983,2.840)**
Inconsistency measures: Not applicable
Incoherence judgment No concerns

Comparison **ARNI:Digoxin**
Evidence: indirect
Indirect odds ratio: **0.983(0.681,1.419)**
Inconsistency measures: Not applicable
Incoherence judgment No concerns

Comparison **ARNI:MRA**
Evidence: indirect
Indirect odds ratio: **1.079(0.810,1.438)**
Inconsistency measures: Not applicable
Incoherence judgment No concerns

Comparison **ARNI:Placebo**
Evidence: indirect
Indirect odds ratio: **0.982(0.789,1.223)**
Inconsistency measures: Not applicable
Incoherence judgment No concerns

Comparison **ARNI:SGLT-2**
Evidence: indirect
Indirect odds ratio: **1.044(0.802,1.359)**
Inconsistency measures: Not applicable
Incoherence judgment No concerns

Comparison **ARNI:Vericiguat 10mg**
Evidence: indirect
Indirect odds ratio: **0.444(0.173,1.136)**
Inconsistency measures: Not applicable
Incoherence judgment No concerns

Comparison **ARNI:Vericiguat 15mg**
Evidence: indirect
Indirect odds ratio: **0.685(0.251,1.872)**
Inconsistency measures: Not applicable
Incoherence judgment No concerns

Comparison **Beta blockers:Digoxin**
Evidence: indirect
Indirect odds ratio: **0.588(0.334,1.036)**
Inconsistency measures: Not applicable
Incoherence judgment No concerns

Comparison **Beta blockers:MRA**
Evidence: indirect
Indirect odds ratio: **0.646(0.385,1.084)**
Inconsistency measures: Not applicable
Incoherence judgment No concerns

Comparison **Beta blockers:SGLT-2**
Evidence: indirect
Indirect odds ratio: **0.625(0.377,1.036)**
Inconsistency measures: Not applicable
Incoherence judgment No concerns

Comparison **Beta blockers:Vericiguat 10mg**
Evidence: indirect
Indirect odds ratio: **0.266(0.095,0.747)**
Inconsistency measures: Not applicable
Incoherence judgment No concerns

Comparison **Beta blockers:Vericiguat 15mg**
Evidence: indirect
Indirect odds ratio: **0.410(0.137,1.224)**
Inconsistency measures: Not applicable
Incoherence judgment No concerns

Comparison **Digoxin:MRA**
Evidence: indirect
Indirect odds ratio: **1.098(0.775,1.555)**
Inconsistency measures: Not applicable
Incoherence judgment No concerns

Comparison **Digoxin:SGLT-2**
Evidence: indirect
Indirect odds ratio: **1.062(0.764,1.477)**
Inconsistency measures: Not applicable
Incoherence judgment No concerns

Comparison **Digoxin:Vericiguat 10mg**
Evidence: indirect
Indirect odds ratio: **0.452(0.173,1.180)**
Inconsistency measures: Not applicable
Incoherence judgment No concerns

Comparison **Digoxin:Vericiguat 15mg**
Evidence: indirect
Indirect odds ratio: **0.697(0.250,1.941)**
Inconsistency measures: Not applicable
Incoherence judgment No concerns

Comparison **MRA:SGLT-2**
Evidence: indirect
Indirect odds ratio: **0.968(0.764,1.226)**
Inconsistency measures: Not applicable
Incoherence judgment No concerns

Comparison **MRA:Vericiguat 10mg**
Evidence: indirect
Indirect odds ratio: **0.411(0.162,1.046)**
Inconsistency measures: Not applicable
Incoherence judgment No concerns

Comparison **MRA:Vericiguat 15mg**
Evidence: indirect
Indirect odds ratio: **0.635(0.234,1.723)**
Inconsistency measures: Not applicable
Incoherence judgment No concerns

Comparison **SGLT-2:Vericiguat 10mg**
Evidence: indirect

Indirect odds ratio: **0.425(0.168,1.073)**
Inconsistency measures: Not applicable

Incoherence judgment No concerns

Comparison **SGLT-2:Vericiguat 15mg**
Evidence: indirect

Indirect odds ratio: **0.656(0.243,1.769)**
Inconsistency measures: Not applicable

Incoherence judgment No concerns
