# Supplementary material for: Effect of pharmacological treatment on outcomes of heart failure with preserved ejection fraction: an updated systematic review and network meta-analysis of randomized controlled trials
Source: Cardiovasc Diabetol. 2022 Nov 8;21:237. doi: 10.1186/s12933-022-01679-2 (PMC9644566; doi:10.1186/s12933-022-01679-2)
Supplement: Supplementary file 6 — Additional file 6. Overal evaluattion of the network meta-analysis. [file 12933_2022_1679_MOESM6_ESM.pdf]

Confidence In Network Meta Analysis - CINeMA 2.0.0 - Project: **HFpEF network meta-analysis**

HFpEF network meta-analysis

Comparison	Number of Studies	Within-study bias	Reporting bias	Indirectness	Imprecision	Heterogeneity	Incoherence	Confidence rating	Reason(s) for downgrading
Mixed evidence									
**ACEI vs ARB**	1	Major concerns ☐	Some concerns ☐	No concerns	Some concerns ☐	No concerns	Some concerns ☐	Moderate	|
**ACEI vs Placebo**	3	Major concerns ☐	Some concerns ☐	No concerns	Major concerns ☐	No concerns	Some concerns ☐	High	|
**ARB vs ARNI**	2	No concerns	Low risk	Some concerns ☐	Major concerns ☐	No concerns	No concerns	Low	|
**ARB vs Placebo**	2	No concerns	Low risk	No concerns	No concerns	No concerns	No concerns	Low	|
**Beta blockers vs Placebo**	2	No concerns	Low risk	No concerns	No concerns	Major concerns ☐	No concerns	Low	|
**Digoxin vs Placebo**	1	Major concerns ☐	Low risk	No concerns	No concerns	No concerns	Major concerns ☐	High	|

Comparison	Number of Studies	Within-study bias	Reporting bias	Indirectness	Imprecision	Heterogeneity	Incoherence	Confidence rating	Reason(s) for downgrading
**MRA vs Placebo**	2	No concerns	Low risk	No concerns	No concerns	No concerns	No concerns	Low	
**Placebo vs SGLT-2**	1	No concerns	Low risk	Major concerns ☐	No concerns	No concerns	No concerns	Moderate	
**Placebo vs Vericiguat 10mg**	1	Some concerns ☐	Low risk	No concerns	No concerns	No concerns	No concerns	Low	
**Placebo vs Vericiguat 15mg**	1	Some concerns ☐	Low risk	No concerns	No concerns	No concerns	No concerns	Low	
**Vericiguat 10mg vs Vericiguat 15mg**	1	Some concerns ☐	Low risk	No concerns	No concerns	No concerns	No concerns	Low	
Indirect evidence									
**ACEI vs ARNI**	--	No concerns	Some concerns ☐	Major concerns ☐	No concerns	No concerns	No concerns	Moderate	
**ACEI vs Beta blockers**	--	No concerns	Low risk	No concerns	No concerns	No concerns	Some concerns ☐	Low	
**ACEI vs Digoxin**	--	Some concerns ☐	Low risk	No concerns	No concerns	No concerns	No concerns	Low	

Comparison	Number of Studies	Within-study bias	Reporting bias	Indirectness	Imprecision	Heterogeneity	Incoherence	Confidence rating	Reason(s) for downgrading
**ACEI vs MRA**	--	No concerns	Low risk	No concerns	No concerns	No concerns	No concerns	Low	|
**ACEI vs SGLT-2**	--	No concerns	Low risk	No concerns	No concerns	No concerns	No concerns	Low	|
**ACEI vs Vericiguat 10mg**	--	Some concerns ☐	Low risk	No concerns	No concerns	No concerns	No concerns	Low	|
**ACEI vs Vericiguat 15mg**	--	Some concerns ☐	Low risk	No concerns	No concerns	No concerns	No concerns	Low	|
**ARB vs Beta blockers**	--	No concerns	Low risk	No concerns	No concerns	Major concerns ☐	No concerns	Moderate	|
**ARB vs Digoxin**	--	Some concerns ☐	Low risk	No concerns	No concerns	No concerns	No concerns	Low	|
**ARB vs MRA**	--	No concerns	Low risk	No concerns	No concerns	No concerns	No concerns	Low	|
**ARB vs SGLT-2**	--	No concerns	Low risk	No concerns	No concerns	No concerns	No concerns	Low	|
**ARB vs Vericiguat 10mg**	--	Major concerns ☐	Low risk	No concerns	No concerns	No concerns	No concerns	Moderate	|

Comparison	Number of Studies	Within-study bias	Reporting bias	Indirectness	Imprecision	Heterogeneity	Incoherence	Confidence rating	Reason(s) for downgrading
**ARB vs Vericiguat 15mg**	--	Major concerns ☐	Low risk	No concerns	No concerns	No concerns	No concerns	Moderate	|
**ARNI vs Beta blockers**	--	No concerns	Low risk	No concerns	No concerns	No concerns	No concerns	Low	|
**ARNI vs Digoxin**	--	Some concerns ☐	Low risk	No concerns	No concerns	No concerns	No concerns	Low	|
**ARNI vs MRA**	--	No concerns	Low risk	No concerns	No concerns	No concerns	No concerns	Low	|
**ARNI vs Placebo**	--	No concerns	Low risk	No concerns	No concerns	No concerns	No concerns	Low	|
**ARNI vs SGLT-2**	--	No concerns	Some concerns ☐	No concerns	No concerns	No concerns	No concerns	Low	|
**ARNI vs Vericiguat 10mg**	--	No concerns	Low risk	No concerns	No concerns	No concerns	No concerns	Low	|
**ARNI vs Vericiguat 15mg**	--	No concerns	Low risk	No concerns	No concerns	No concerns	No concerns	Low	|

Comparison	Number of Studies	Within-study bias	Reporting bias	Indirectness	Imprecision	Heterogeneity	Incoherence	Confidence rating	Reason(s) for downgrading
**Beta blockers vs Digoxin**	--	Some concerns ▢	Low risk	No concerns	No concerns	No concerns	No concerns	Low	|
**Beta blockers vs MRA**	--	No concerns	Low risk	No concerns	No concerns	No concerns	No concerns	Low	|
**Beta blockers vs SGLT-2**	--	No concerns	Low risk	No concerns	Major concerns ▢	No concerns	No concerns	Moderate	|
**Beta blockers vs Vericiguat 10mg**	--	Some concerns ▢	Low risk	No concerns	No concerns	Major concerns ▢	No concerns	Moderate	|
**Beta blockers vs Vericiguat 15mg**	--	Some concerns ▢	Low risk	No concerns	No concerns	No concerns	No concerns	Low	|
**Digoxin vs MRA**	--	Some concerns ▢	Low risk	No concerns	No concerns	No concerns	No concerns	Low	|
**Digoxin vs SGLT-2**	--	Some concerns ▢	Low risk	No concerns	No concerns	No concerns	No concerns	Low	|

Comparison	Number of Studies	Within-study bias	Reporting bias	Indirectness	Imprecision	Heterogeneity	Incoherence	Confidence rating	Reason(s) for downgrading
**Digoxin vs Vericiguat 10mg**	--	Some concerns ☐	Low risk	No concerns	No concerns	No concerns	No concerns	Low	
**Digoxin vs Vericiguat 15mg**	--	Some concerns ☐	Low risk	No concerns	No concerns	No concerns	No concerns	Low	
**MRA vs SGLT-2**	--	No concerns	Low risk	No concerns	No concerns	No concerns	No concerns	Low	
**MRA vs Vericiguat 10mg**	--	Some concerns ☐	Low risk	No concerns	No concerns	No concerns	No concerns	Low	
**MRA vs Vericiguat 15mg**	--	Some concerns ☐	Low risk	No concerns	No concerns	No concerns	No concerns	Low	
**SGLT-2 vs Vericiguat 10mg**	--	Some concerns ☐	Low risk	No concerns	No concerns	No concerns	No concerns	Low	
**SGLT-2 vs Vericiguat 15mg**	--	Some concerns ☐	Low risk	No concerns	No concerns	No concerns	No concerns	Low	
